# Cardiac cavernous hemangioma with high fluorodeoxyglucose uptake on preoperative positron emission tomography/computed tomography: a case report

**DOI:** 10.1186/s44215-023-00054-1

**Published:** 2023-07-10

**Authors:** Takuma Kobayashi, Satoshi Numata, Yu Hohri, Hidetake Kawajiri, Hitoshi Yaku

**Affiliations:** Department of Cardiovascular Surgery, Kyoto Prefectural University of Medicine, Kyoto, Japan

**Keywords:** Atrial appendage, Heart neoplasms, Magnetic resonance imaging

## Abstract

**Background:**

Reports on cardiac hemangioma detection by F-18-deoxyglucose (FDG) positron emission tomography (PET)/computed tomography (CT) are extremely rare.

**Case presentation:**

Here we describe a case of a 61-year-old man with hemangioma. CT revealed a tumor with a size of 60 mm. Magnetic resonance imaging showed high signal intensity on T2-weighted images. Hemangioma was initially suspected. However, PET/CT showed high FDG absorption, which led us to suspect a malignant tumor. The tumor was resected, and a cavernous hemangioma diagnosis was made by postoperative histopathological examination.

**Conclusions:**

This case may serve as a reference for clinicians to become more aware of the potential application of FDG PET/CT for cardiac hemangioma detection.

## Background

Cardiac hemangiomas are rare benign cardiac tumors that comprise approximately 2% of resected cardiac tumors. They can occur at any age and develop anywhere in the heart [1]. Although cardiac hemangiomas almost always arise within the heart, very few reports of hemangiomas arising in the left atrial appendage exist [2].

Here, we report a case of hemangioma arising in the left atrial appendage, which was preoperatively suspected to be malignant due to a high level of F-18-deoxyglucose (FDG) uptake on positron emission tomography (PET)/computed tomography (CT). Postoperatively, we diagnosed the patient with cardiac cavernous hemangioma based on histopathological assessment.

## Case presentation

A 61-year-old healthy man with unremarkable medical and family history was admitted to a local hospital for shoulder pain. Electrocardiography showed ST-segment elevation in leads V2-6. Transthoracic echocardiography revealed mild pericardial effusion. Although coronary angiography revealed no significant coronary artery stenosis, CT revealed a cardiac tumor. Consequently, pericarditis was diagnosed. After his condition stabilized, he was referred to our hospital for surgical treatment.

**Fig. 1:**
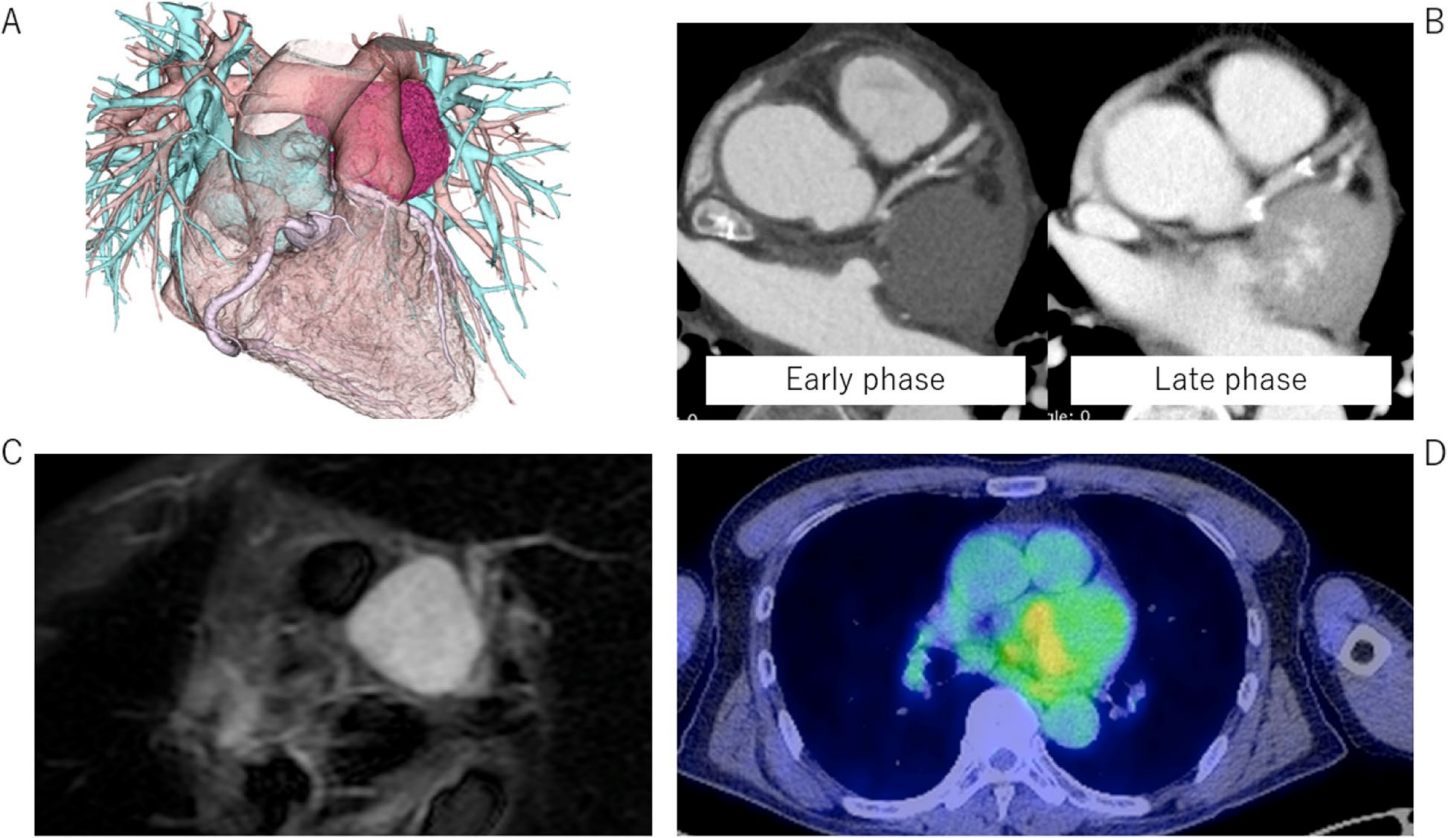
**A** Preoperative computed tomography (CT) imaging showed a tumor with a size of approximately 60 mm. **B** The tumor was located between the left atrium and epicardium. Contrast medium spread more widely in the tumor in the late phase than in the early phase. **C** The tumor showed high signal intensity on magnetic resonance imaging (MRI) T2-weighted image. **D** The tumor demonstrated high uptake of F-18-deoxyglucose (FDG) on positron emission tomography (PET)/CT (white arrow)

Preoperative echocardiography revealed an isoechoic tumor adjacent to the epicardium. However, it did not develop in the heart chamber. Preoperative CT imaging showed a tumor with a size of approximately 60 mm between the left atrium and epicardium (Fig. 1A). Contrast medium spread more widely in the tumor in the late phase than in the early phase (Fig. 1B). Moreover, the tumor showed high signal intensity on magnetic resonance imaging (MRI) T2-weighted image (Fig. 1C). However, since the tumor showed high FDG uptake on PET/CT (Fig. 1D), it was suspected to be malignant.

**Fig. 2:**
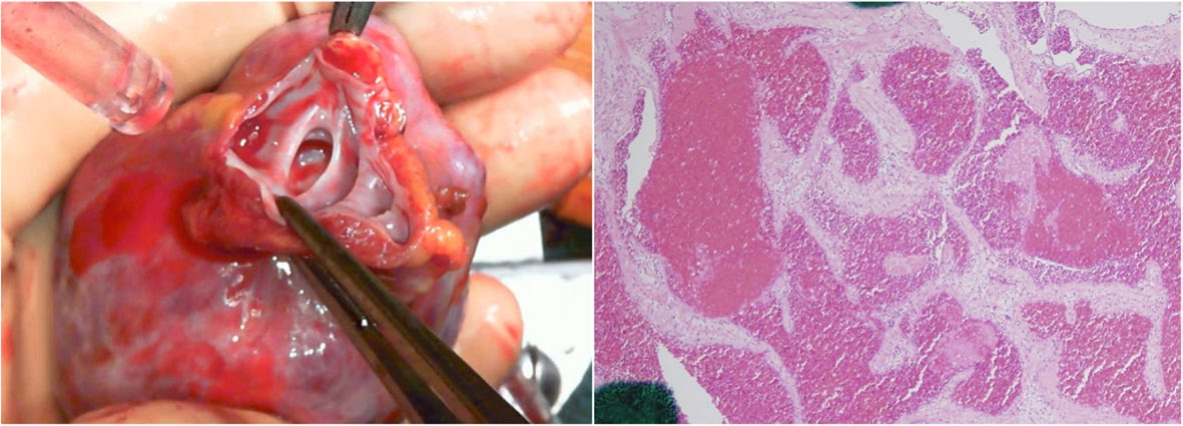
Micropathologic views. Histopathological examination with hematoxylin and eosin staining showed a tumor consisting largely of blood clots with some proliferated capillaries (hematoxylin, magnification × 4)

We performed tumor resection via median full sternotomy on cardiopulmonary bypass. During surgery, we found that the tumor had a smooth surface and was connected to the left atrial appendage. Adhesions were not observed around the pericardial cavity. It was resected using a left atrial appendage. The patient was diagnosed with cardiac cavernous hemangioma based on histopathological assessment (Fig. 2). The patient was discharged 12 days after surgery without any complications or additional treatment requirements, such as chemotherapy or radiotherapy. Four months after surgery, the patient was in good condition without recurrence.

Written informed consent was obtained from the patient for the publication of this case report and the accompanying images.

## Discussion and conclusions

Cardiac tumors are rare tumors that present with a variety of symptoms and outcomes, depending on the type and location of the tumor. While early diagnosis and treatment of benign tumors is usually curative, distinguishing these tumors from malignant disease is important. In previous literature, angiosarcoma, rhabdomyosarcoma, and mesothelioma were the most frequent primary malignant diseases of the heart [3]. Hemangiosarcomas, rhabdomyosarcomas, and mesotheliomas are considered to have predilections for the right atrium, the ventricles, and the epicardium, respectively. While most cardiac hemangiomas occur within the heart, there have been very few reports of hemangiomas arising within the left atrial appendage. We considered it important to differentiate hemangiomas from malignant tumors preoperatively and attempted to make a diagnosis using FDG PET-CT.

The majority of hemangiomas are discovered incidentally or when spontaneous rupture results in hemorrhage or hemorrhagic pericardial effusion. Hemangiomas are also known to cause non-hemorrhagic pericardial effusions. However, tumor bleeding can also cause pericardial tamponade. Rupture and death due to conduction disturbances and tamponade can occur [4, 5].

Diagnosis is usually made using imaging modalities, such as echocardiography, CT, MRI, and coronary angiography. Echocardiography, a real-time imaging modality, has become the most important screening and diagnostic tool due to its relatively high accuracy and non-invasive nature.

Enhanced MRI and CT have been used to assess the size, location, and extracardiac involvement of cardiac cavernous hemangiomas. Coronary angiography is required to assess the presence of nutrient vessels and tumor blush in patients with cardiac cavernous hemangiomas. Tumor blush is a classic sign of hemangiomas. Although it has been detected in approximately one-third (34.4%) of patients with cardiac cavernous hemangioma [6], tumor blush was not observed in our case.

Echocardiography, CT, MRI, and angiography have been used to detect and evaluate cardiac tumors, including cardiac hemangiomas. Therefore, imaging findings of such tumors using these modalities are relatively well established in the literature. However, data on the use of FDG PET/CT to detect cardiac hemangiomas are very limited. Due to hyperaccumulation on FDG-PET, which was suggestive of malignancy, we decided to perform surgery. However, the patient was histopathologically diagnosed with hemangioma.

Hemangioma in this case showed hyperaccumulation on FDG-PET, which was thought to result from hemorrhage and inflammation within the tumor. Similar findings have also been reported in ectopic hemangiomas, and we believe that intratumoral hemorrhage- or inflammation-associated hyperaccumulation on FDG PET/CT may be a characteristic imaging finding in hemangiomas, despite their benign nature [7].

Although preoperative diagnosis is difficult, when hemangioma or malignancy is suspected or when extension into the pericardial sac is observed, aggressive treatment should be considered even for benign tumors to avoid pericardial tamponade due to tumor bleeding. This rare case of cardiac hemangioma was evaluated with PET/CT and showed FDG uptake. Therefore, this finding should be incorporated into the literature regarding benign tumors with FDG uptake and be considered during the interpretation of PET/CT studies.

## Data Availability

Data of this case are available in the article.

## Abbreviations

CT: Computed tomography
FDG: F-18-deoxyglucose
MRI: Magnetic resonance imaging
PET: Positron emission tomography
